# Suboptimal Urinary Iodine Level in Pregnant Women from Perimarine Area of Romania

**DOI:** 10.3390/jcm14113666

**Published:** 2025-05-23

**Authors:** Olesea Scrinic, Eduard Circo, Delia Corina, Seila Musledin

**Affiliations:** 1 Endocrinology Department, Faculty of Medicine, “Ovidius” University of Constanta, 900527 Constanta, Romania; 2 The National Institute for Mother and Child Health “Alessandrescu-Rusescu”, 020395 Bucharest, Romania

**Keywords:** pregnant, urinary iodine, perimarine, thyroid

## Abstract

**Objective**: Maintaining adequate iodine intake during pregnancy contributes to achieving a viable fetus with proper neuropsychological development. Because of the lack of national data regarding the assessment of iodine status in pregnant women—conducted through urinary iodine determination in perimarine regions, geographical areas characterized by sufficient iodine intake—this study was undertaken. **Materials and Methods**: The study evaluated iodine status in a cohort of pregnant women from southeastern region of Romania, perimarine area, assessing iodine intake indicators and the severity of iodine deficiency levels. **Results**: Iodine nutritional status, based on urinary iodine concentration values adjusted to urinary creatinine, was insufficient in 47.3% of pregnancies. Moderate iodine deficiency was found in 43.2%, while severe iodine deficiency was identified in only 4.1%. **Conclusions**: Although this is a non-endemic region for iodine deficiency disorders, the perimarine area of Romania presents a moderate iodine deficiency among special population groups such as pregnant women. This situation necessitates iodine intake adaptation and periodic monitoring to prevent maternal-fetal thyroid dysfunctions.

## 1. Introduction

Adequate iodine intake during pregnancy is fundamental for thyroid hormone synthesis and secretion, which plays a crucial role in the neurocognitive development of the fetus. Iodine deficiency leads to a wide range of adverse health effects due to inadequate thyroid hormone production, commonly known as iodine deficiency disorders (IDDs).

A physiological adaptation of the thyroid gland occurs during pregnancy, but if iodine intake is insufficient or excessive, these normal adaptations can turn into pathological changes [1]. During pregnancy, iodine intake undergoes a series of concentration changes due to the increased requirement for maintaining maternal euthyroidism, the transfer of iodine to the fetus, as well as the increase in maternal renal iodine clearance, leading to an increased iodine requirement [2], also renal blood flow increases and the glomerular filtration rate rises by approx. 40–50%, which is the reason for the increase in 24-h urinary volume, causing urinary dilution and therefore increasing renal iodine clearance and renal iodine excretion [3].

In cases of iodine deficiency, intrathyroidal iodine reserves decrease, hypothyroxinemia occurs, followed by an increase in TSH and thyroid hypertrophy [4]. The daily iodine requirement is regulated by WHO, together with ICCIDD and UNICEF, adapted to specific population categories: pregnant/lactating women—250 mcg/L, women of reproductive age (15–49 years)—150 mcg/L, children < 2 years—90 mcg/L, school-aged children (6–12 years)—120 mcg/L, adolescents (>12 years) and adults—150 mcg/L [5].

The assessment of iodine nutritional status is performed by evaluating indicators of adequate or insufficient intake within a population, necessary for monitoring iodine deficiency disorders and the effectiveness of universal salt iodization programs recommended by WHO/UNICEF and IGN (Iodine Global Network). The evaluated indicators are: goiter prevalence—assessed by palpation or ultrasound (evaluated in school-aged children), median urinary iodine concentration (mUIC) (evaluated in school-aged children and pregnant women), neonatal serum TSH (thyroid- stimulating hormone) levels, and serum thyroglobulin levels (evaluated in school-aged children) [5].

Even though iodine deficiency was considered to be the prerogative of mountainous regions and alluvial plains at high altitudes and a considerable distance from the sea, the frequent use of iodine intake indicators such as UIC has also detected iodine deficiency in perimarine and coastal areas or even in areas previously declared free of iodine deficiency. Adequate iodine intake, assessed according to the median urinary iodine concentration in pregnant women, is within the range of 150–249 mcg/L, more than adequate: 250–499 mcg/L, insufficient <150 mcg/L, and excessive >500 mcg/L, while for school-aged children >100 mcg/L is considered an adequate iodine intake (according to WHO/UNICEF) [5].

The 2017 reports from IGN presented, for the first time, data on the estimated global iodine status for pregnant women. Epidemiological information on pregnant women was provided by 72 (from 194), with data of insufficient iodine intake in 39 of the countries [6].

At the national level in Romania, one of the most recent studies conducted by National Institute for Mother and Child Health “Alessandrescu—Rusescu” Bucharest (NIMCH) and the National Institute of Endocrinology “IC Parhon” Bucharest in 2016–2017, on a cohort of 665 pregnant women from 15 endemic counties of Romania, regarding the assessment of iodine status in pregnancy still reveals the persistence of suboptimal urinary iodine values (median UIC—116 mcg/L) in pregnant women from endemic areas of Romania [7]. 

Recent statistical data provided by the Iodine Global Network in 2024 regarding global iodine status among school-aged children, have highlighted a significant reduction in the number of countries with insufficient iodine intake, from 54 in 2003 to 20 in 2023, out of a total of 194 WHO member countries [8].

Perimarine areas are geographical regions protected from iodine deficiency, therefore, Dobrogea, the perimarine region of Romania, is considered a territory with sufficient iodine intake. However, certain population categories, such as pregnant women, children, and breastfeeding women, have increased iodine intake requirements in different and variable amounts, necessitating adaptation in accordance with international and national regulations.

The present study aimed to evaluate the nutritional iodine status and the degrees of iodine deficiency in a vulnerable and special category composed of pregnant women originating from Dobrogea, the southeastern region of Romania, located between the Danube and the Black Sea.

## 2. Materials and Methods

A prospective clinical study was conducted on a number of 82 pregnant women in the first and second trimester of pregnancy, originating from the perimarine area of Romania, carried out in the period 2020–2022. Following the application of the inclusion criteria (confirmed pregnancy, origin and residence in Dobrogea, pregnancy in the first/second trimester) and exclusion criteria (pregnant women who had not given birth in Romania, pregnant women with extreme urinary iodine values > 800 mcg/mL, and lack of informed consent), 74 pregnant women (90%) were enrolled, while 8 pregnant women (10%) were excluded from the study for the following reasons: 2 pregnant women identified with extreme urinary iodine values (samples considered contaminated with iodine), another 6 pregnant women had not given birth on Romanian territory.

According to the statistical yearbook of Constanta County, the total resident population in 2020 was 670,202 and in 2021—662,124, and the number of newborns in 2020 was 6868 and in 2021 was 6551.

Methodology for determining urinary iodine: The determination of urinary iodine concentration or urinary iodine levels in the pregnant women included in the study was carried out within the National Institute for Mother and Child Health “Alessandrescu-Rusescu”—Bucharest, iodine laboratory. The laboratory is subject to external quality control through the EQUIP (Programme for Ensuring the Quality of Iodine Procedures) program led by the Centers for Disease Control (CDC, USA) and since 2017, it has received recognition for successful participation in the evaluation (1-EQUIP), furthermore, the laboratory is part of the project aimed at standardizing the measurement of urinary iodine throughout Europe [9]. Samples were collected from spontaneous urine (first/second trimester of pregnancy), using disposable cups, urine monovetes and then stored at −18% in the refrigerator and subsequently transported in a refrigerated container to National Institute for Mother and Child Health “Alessandrescu—Rusescu” Bucharest.

Method for determining urinary iodine: digestion with ammonium persulfate followed by the Sandell-Kolthoff reaction. The determination of iodine concentration in urine is based on spectrophotometric measurement of a colorimetric complex at 420 nm, the complex being formed based on the Sandell-Kolthoff reaction [10]. Severity grades of iodine deficiency based on iodine concentration in pregnant women: severe deficiency—below 50 mcg/L; moderate deficiency—between 50 and 149 mcg/L; absence of deficiency—between 150–499 mcg/L; excess—above 500 mcg/L [11].

Determination of urinary creatinine: Method for determining urinary creatinine: Jaffe Method.

Following the obtaining of urinary iodine concentration (UIC) in mcg/L and urinary creatinine (CrU) in g/L, the ratio between urinary iodine concentration and urinary creatinine (UIC/CrU ratio) was calculated, considered a more reliable indicator of iodine excretion and implicitly iodine intake in the population of pregnant women from a geographical area, since the increase in glomerular filtration rate and 24-h urinary volume induces urinary dilution with increased renal clearance of iodine [12,13]. In this context, the UIC/CrU ratio can be an optimal index for assessing iodine nutritional status in pregnant women, under conditions that exclude variations in volume and urinary dilution, it also better reflects urinary iodine excretion in 24 h and as well as circulating serum iodine levels [5,14].

### Statistical Data Analysis

The research data were analyzed and processed using the IBM SPSS Statistics 23 statistical program. The tests used were: descriptive statistics (for characterizing discrete and continuous variables defined at the database level), graphs, non-parametric statistical tests—addressed to categorical variables (χ^2^ association test). Non-parametric statistical tests were used for ordinal data or addressed to numerical variables (Mann-Whitney U Test, Median Test, Kruskal-Wallis Test). Statistical significance was considered at *p* < 0.05 [15].

## 3. Results

The median age of the 74 pregnant women included in the study was 29 years, and the gestational age was 11 weeks.

The majority of pregnant women came from an urban environment—80.2% (n = 61) from the municipalities of Constanța and Tulcea. Slightly more than half of the cases were multiparous and the average BMI fell within the normal weight rang. A percentage of 40.5% (n = 30) of the pregnant women were smokers.

A personal history of thyroid disorders before pregnancy was present in 30% (n = 21) of the pregnant women, while a family history of thyroid disorders was found in 39% (n = 29). Although insufficient, only 82% (n = 61) of the pregnant women reported consuming iodized salt in their daily diet and only 33.7% (n = 25) consumed iodine-containing supplements during pregnancy (Table 1).

### Evaluation of Maternal Iodine Status

The assessment of median UIC mcg/L as an indicator of iodine intake recommended by WHO revealed a value of 133.03 mcg/L (IQR: 69.11–204.65 mcg/L) corresponding to an insufficient iodine intake of the pregnant women, while the median UIC/UCr ratio indicated a value of 157.08 µg/g, characteristic of an adequate iodine intake.

The determination of nutritional iodine status based on UIC in the group of studied pregnant women identified an inadequate intake—42 cases (56.7%) and adequate intake—32 cases (43.24%), however, the use of a more reliable and sensitive instrument, such as the UIC/UCr ratio, evaluates insufficient iodine intake in 35 cases (47.3%) and adequate intake in 39 cases (52.7%).

The distribution of pregnant women according to the urinary iodine value based on the severity degree of iodine deficiency identified the following results according to UIC: severe deficiency (<50 mcg)—16.2%, moderate deficiency (50–150 mcg/L)—40.5%, and no iodine deficiency—43.2%, while according to the UIC/UCr ratio value: severe deficiency—4.1%, moderate deficiency—43.2%, no iodine deficiency—52.7% (Table 2).

In Table 3, the comparative assessment between trimesters reveals an increase in UIC and UIC/UCr in the second trimester compared to the first trimester, but without statistically significant differences.

Pregnant women who declared the consumption of iodized salt had a median UIC of 134.7 mcg/L (IQR: 65.2–205.8 mcg/L), while those without iodized salt consumption had a median UIC of 130 mcg/L (IQR: 96.3–254.9 mcg/L), without statistically significant differences between the median UIC values among the two analyzed categories (Independent Sample Median test: *p* = 0.541 > α = 0.05).

Iodine-containing supplements (tablets containing at least 150 mcg daily) were consumed by 25 pregnant women (33.7%), with a median UIC value of 198.1 mcg/L (IQR: 123.2–340.4 mcg/L), while the rest of the pregnant women without iodine supplement consumption had a median UIC of 115 mcg/L (IQR: 55.9–180.05 mcg/L), with statistically significant differences between the two categories of patients (*p* = 0.014).

Additionally, the median UIC/UCr in the subgroup with iodine supplement consumption was 206.5 mcg/g and 133 mcg/g in the subgroup without iodine supplement consumption, with a statistically significant difference between the two subgroups (*p* = 0.049).

We observed statistically significant differences between the distribution of UIC values (Independent Sample Mann-Whitney U test: *p* = 0.001), as well as UIC/UCr ratio (mcg/L) (*p* = 0.005) in the category of pregnant women using iodine supplements compared to those not consuming iodine supplements.

**Figure 1:**
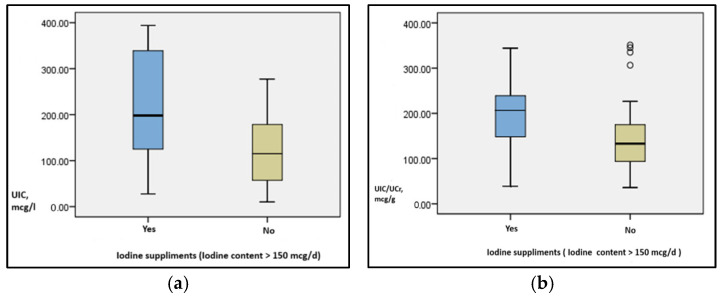
Distribution of urinary iodine concentration (mcg/L) (**a**) and the UIC/UCr ratio, mcg/g (**b**) according to the consumption of iodine supplements.

Additionally, we identified statistically significant differences between the median UIC values (Independent Sample Median test: *p* = 0.014), as well as the UIC/UCr ratio (mcg/L) (*p* = 0.049) (Figure 1).

## 4. Discussion

The motivation for this study started from the lack of information and data regarding the nutritional iodine status of pregnant women originating from areas without a declared iodine deficiency, namely the perimarine area of Romania, the Dobrogea region. National population studies have presented a series of results from regions considered endemic for iodine deficiency, most of them being conducted on pregnant women in the last trimester of pregnancy.

The spectrum of thyroid disorders in iodine-sufficient areas is characterized by the prevalence of autoimmune thyroid diseases, and under pregnancy conditions, they require periodic monitoring with the initiation or adjustment of appropriate therapy [2].

Although the Dobrogea region is characterized as a non-endemic area for iodine deficiency, iodine intake under conditions of increased iodine needs, such as pregnancy, may present various variabilities depending on numerous associated factors. The use of iodized or non-iodized salt, iodine-containing supplements, the consumption of marine-origin foods are just some of the elements that can influence iodine nutritional status.

The originality of the present study may be considered the fact that it is the only study in the last 25 years that evaluates the iodine status of pregnant women at the beginning of pregnancy in the perimarine area of Romania.

The epidemiological criteria recommended by WHO/UNICEF/ICCIDD for evaluating nutritional iodine intake in pregnant women is based on the median urinary iodine concentration (UIC) and is considered insufficient <150 μg/L and adequate >150 μg/L [5].

The median urinary iodine concentration for the entire studied group of pregnant women was below 150 mcg/mL, a value characteristic of insufficient iodine intake. The values obtained are very close to those of the study conducted by the National Institute of Endocrinology “IC Parhon” Bucharest and INSMC “Alfred Rusescu” in 2016–2017 at the national level on a population of 665 pregnant women originating from endemic areas of Romania, where they obtained a median UIC of 116 mcg/L specific to insufficient iodine intake [7].

Similar results are presented by E. Limbert et al. in 2010 on the pregnant women population (3631 cases) originating from the coastal and insular area of Portugal, proving insufficient iodine intake [16].

Although only the mountainous and peri-mountainous regions of Romania were considered endemic regarding iodine deficiency in the past, according to the largest studies in 2005 (1595 pregnant women) and 2014 (8803 newborns) conducted after the introduction of USI starting in 2002, iodine deficiency in pregnant women is found, although of mild severity, across the entire territory of Romania [17,18].

However, according to the most recent data from 2024 from the Iodine Global Network, the general population of Romania is characterized by an adequate iodine intake based on urinary iodine determination, with a median UIC in school-aged children of 255 mcg/L, but for pregnant women, iodine intake is insufficient with a median UIC of 131 mcg/L [19]. 

The study conducted by Zimmermann et al. and published in The Lancet in 2015 con-firms Romania as a country with insufficient iodine intake among pregnant women based on the median urinary iodine concentration [11].

Thus, more than 20 years after the implementation of USI in Romania, pregnant women continue to present an iodine deficiency and the possible causes of inadequate iodine status for pregnant women in the perimarine region of Romania may be the reduction in salt intake among pregnant women or insufficient iodization of salt, or inadequate consumption of iodized salt associated with a period of increased iodine requirement to maintain normal thyroid hormone levels throughout pregnancy, also inadequate preconception iodine status may have an influence.

The perimarine area of Romania also presents a series of geological causes, such as the predominance of limestone rocks and alkaline salts in the chemical composition of the Dobrogea soil, and groundwater being highly mineralized due to calcium salts with an alkaline reaction. The main characteristic of the soil in which limestone rocks predominate is the low iodine content, however, this phenomenon is balanced by the geographical location of Dobrogea where the marine iodine source is ensured in the soil through rainwater. Thus, particular conditions of interference of calcium salts with intestinal absorption of iodides, individual reactivity, regional quantitative variations of iodine, but also the use of non-iodized salt may be involved in the differences in iodine intake in the general population and that of pregnant women [20].

The increase in the glomerular filtration rate during pregnancy by approximately 50% and in the 24-h urinary volume is also associated with increased renal iodine excretion, thus several studies have analyzed the use of a more optimal indicator of iodine nutritional status during pregnancy, minimizing variations caused by differences in urinary volume and dilution, this being the ratio between urinary iodine concentration and urinary creatinine, compared in specificity to the circulating serum iodine level [5,16,17].

In our study, the median UIC/UCr ratio was 152.83 mcg/g, thus exceeding only slightly the reference threshold of 150 mcg/g characteristic of adequate iodine intake for pregnant women. The proportion of insufficient iodine intake among pregnant women in the study varies depending on the epidemiological indicator used, according to UIC—56.7% and to UIC/UCr ratio—47.3%, thus the evaluation based solely on UIC reveals a prevalence 9.4% higher than the evaluation based on the UIC/UCr ratio of insufficient iodine intake, overestimating iodine deficiency in pregnancy.

The distribution of the severity degrees of iodine deficiency based on UIC compared to the UIC/UCr ratio also presents differences in the present study, with a higher prevalence of severe iodine deficiency reported to urinary iodine (<50 mcg/L) identified in 16.2%, a prevalence that significantly decreases, however, after adjusting urinary iodine to urinary creatinine—4.1%. Moderate iodine deficiency according to UIC—40.5% of cases, a prevalence that, however, increases to 43.2% when adapted to the UIC/UCr ratio. The same results are identified in studies conducted on pregnant women from perimarine areas with an increased prevalence of severe and moderate iodine deficiency in countries such as Portugal, France, or China [21,22]. In Belgium, a study conducted in 1995 on 2000 pregnant women from Brussels, Glinoer et al. identified 56% of cases with a median UIC value < 40 mcg/L and only 10% between 81–160 mcg/L [23]. At the national level, the results were slightly different in the study conducted by Ursu et al. in 2016, carried out on pregnant women from endemic and non-endemic areas, where severe iodine deficiency was found in only 1.7% of cases and moderate iodine deficiency described in only 28%, however, all were evaluated in the last trimester of pregnancy compared to the present study conducted in the first 2 trimesters of pregnancy [24].

We identified an increase in the median UIC/UCr ratio in the second trimester (173 mcg/g) compared to the first trimester (138 mcg/g), a result also described in studies conducted in regions without iodine deficiency (China) or with more than adequate intake (Japan), which support a gradual increase in the UIC/UCr ratio as pregnancy progresses [3,25]. A similar evolution was also identified for the median UIC values, which increase from the first to the second trimester, results also found in studies from Spain [26]. However, there are also studies that support the absence of changes in median UIC with pregnancy progression in France, Sweden, and Sudan [27,28] or even a decrease in urinary iodine during pregnancy due to the progressive depletion of iodine reserves caused by fetoplacental utilization and increased renal filtration not compensated by iodine intake [29,30]. The differences observed in studies between urinary iodine excretion during pregnancy may be explained by ethnic variations in iodine nutritional intake, the severity degrees of iodine deficiency, iodine status and iodine reserves before conception, as well as differences in the structure or size of the studies [31].

A statistically significant association was found between iodine intake based on UIC and pregnant women who consumed iodine supplements during pregnancy (*p* < 0.001). The use of iodine supplements being responsible for the adequate iodine intake of pregnant women. The same significant association was present also between the median UIC and the UIC/UCr ratio and pregnant women with iodine supplement consumption (*p* = 0.014, respectively *p* = 0.049). We did not identify any association between adequate or insufficient iodine intake based on UIC or the UIC/UCr ratio and the modality of iodine intake, whether through iodized salt, iodine supplements, or both modalities.

The determination of UIC according to iodine supplement consumption (prenatal supplements containing iodine of 150 mcg/tablet, the dose used—1 tablet/day) found a normal value for pregnant women with adequate iodine intake, compared to pregnant women without use of iodine supplements who had a much lower UIC value corresponding to insufficient iodine intake.

Significant difference of median distribution of UIC and UIC/UCr ration were found in iodine—suplimented and non suplimented pregnant women in our study. The study conducted by Ursu et al. on pregnant women both from endemic and non-endemic areas did not, however, reveal any statistically significant difference between the subgroups of pregnant women with and without iodine supplement consumption, recording median values of 205.7 mcg/L in pregnant women without iodine supplements and 213.5 mcg/L in pregnant women with iodine supplements, explaining this possibility by the excessive consumption of bakery products where iodized salt is mandatorily used [25].

Although the advantage of administering iodine supplements to pregnant women from areas with severe and moderate iodine deficiency significantly reduces the prevalence of IDD and severe neurological dysfunctions, improves children’s cognitive performance, and decreases perinatal and infant mortality [3,21], it is necessary to individualize iodine supplements in areas without iodine deficiency and especially for pregnant women with thyroid autoimmunity, where excess iodine may also exacerbate thyroid hormone deficiency [32]. From the total number of pregnant women studied, an suboptimal percentage (82.4%) used iodized salt, the remaining 17.5% do not consume salt at all or only non-iodized salt, although the indicator of the efficiency of iodine deficiency disorder prevention programs recommended by WHO is the use of iodized salt in >90% of households [5].

According to the guidelines of international specialty societies, the daily iodine intake during pregnancy must increase to 250 mcg, thus, the administration of prenatal supplements containing iodine between 150 and 200 mcg/daily is recommended both in iodine-deficient and iodine-sufficient areas [32,33]. Only one-third of the pregnant women in our study used iodine supplements (150 mcg/day). WHO supports the individualized use of iodine supplements and for pregnant women in areas where iodized salt is used in more than 90% of households and the median UIC is >100 mcg/L, iodine supplementation is not necessary. For pregnant women in areas where iodized salt use in households is between 20–90%, supplementation with 250 mcg of iodine daily in the form of vitamins or minerals is recommended [34].

## 5. Conclusions

The causes of iodine deficiency in a non-endemic area can be individual pathological conditions concerning iodine bioavailability or failure to ensure intake from dietary sources—foods with low iodine content, confusion between iodized and non-iodized salt, ignoring iodine-containing supplements administered during pregnancy.

Iodine supplementation, necessary for the prevention and treatment of iodine deficiency disorders, must be maintained at a safe level and individualized during pregnancy.

The effectiveness of iodine deficiency prevention programs can be maintained through the association of both iodized salt intake and the use of iodine supplements, even in declared iodine-sufficient areas in the perimarine region of the country, particularly during pregnancy, under conditions of increased iodine requirements.

Following the application of universal salt iodization, under the conditions of a growing population dynamic, with the interference of food sources originating from different regions in terms of mineral composition, nutritional iodine deficiency becomes an individual issue subject to random environmental factors.

Thus, even in geographical areas with adequate iodine content (perimarine area), the possibility of iodine deficiency and thyroid function impairment, particularly in pregnancy, requires periodic population-based investigations of this parameter, with monitoring of iodine status indicators.

## Figures and Tables

**Table 1:** Characteristics and dietary intake data of the pregnant women from perimarine area of Romania.

Variable	
Median age (years), (IQR)	29 (27, 32)
Median of gestational age (IQR)	11 (8, 13)
Multiple pregancy	42 (56, 7%)
Environment:	
-urban	56 (75, 6)
-rural	18 (24, 3)
BMI (kg/m^2^) (IQR)	23.21 (20.44–27.11)
Smoker, n (%)	30 (40, 5)
Family history of thyroid disease, n (%)	29 (39, 1)
Personal history of thyroid disease	21 (30, 4)
Iodised salt intake, n (%)	61 (82, 4)
Iodine containing supplements (>150 mcg/zi), n (%)	25 (33, 7)

**Table 2:** Distribution of pregnant women based on severity degree of iodine deficiency.

Degrees of Severity of Iodine Deficiency	UIC (mcg/L)	UIC/UCr (mcg/g)
	n %	n %
Severe deficiency (<50 mcg/L)	12 16.2	3 4.1
Moderate deficiency (50–149 mcg/L)	30 40.5	32 43.2
Absent deficiency (150–499 mcg/L)	32 43.2	39 52.7

**Table 3:** Determination of urinary iodine concentration depending on trimester, consumption of iodine-containing supplements and iodized salt.

Variabile	UIC, Median	*p*	UIC/UCr, Median	*p*
Trimester				
I	131	0.813	138	0.097
II	147		173	
Iodine supplements				
Yes (n = 25)	198	0.014	206	0.049
No (n = 49)	115		133	
Iodized salt consumption				
Yes (n = 61)	134	0.541	150	1.000
No (n = 13)	130		159	

## Data Availability

All original data and findings discussed in this study are contained within the article. For additional information please contact the corresponding author.

## Abbreviations

The following abbreviations are used in this manuscript:

IGN	Iodine Global Network
CDC	Centers for Disease Control
WHO	World Health Organization
EQUIP	Programme for Ensuring the Quality of Iodine Procedures
ICCIDD	International Council for Control of Iodine Deficiency Disorders
UIC	Urinary iodine concentration
UCr	Urinary creatinine
UNICEF	United Nations International Children’s Emergency Fund
